# The magnitude and associated factors of coagulation abnormalities among liver disease patients at the University of Gondar Comprehensive Specialized Hospital Northwest, Ethiopia, 2022

**DOI:** 10.1186/s12959-023-00479-2

**Published:** 2023-04-03

**Authors:** Abateneh Melkamu, Berhanu Woldu, Chomaw Sitotaw, Masresha Seyoum, Melak Aynalem

**Affiliations:** 1 Department of Medical Laboratory Science, College of Medicine and Health Sciences, Debre Markos University, P.O. Box 269, Debre Markos, Ethiopia; 2 Department of Hematology and Immunohematology, School of Biomedical and Laboratory Sciences, College of Medicine and Health Sciences, University of Gondar, Gondar, Ethiopia

**Keywords:** Activated Partial Thrombin Time, Prothrombin Time, Liver disease, Gondar, Ethiopia

## Abstract

**Background:**

Liver disease is any condition that affects the liver cells and their function. It is directly linked to coagulation disorders since most coagulation factors are produced by the liver. Therefore, this study aimed to assess the magnitude and associated factors of coagulation abnormalities among liver disease patients.

**Methods:**

A cross-sectional study was conducted from August to October 2022 among 307 consecutively selected study participants at the University of Gondar Comprehensive Specialized Hospital. Sociodemographic and clinical data were collected using a structured questionnaire and data extraction sheet, respectively. About 2.7 mL of venous blood were collected and analyzed by the *Genrui CA51* coagulation analyzer. Data were entered into Epi-data and exported to STATA version 14 software for analysis. The finding was described in terms of frequencies and proportions. Factors associated with coagulation abnormalities were analyzed by bivariable and multivariable logistic regression.

**Result:**

In this study, a total of 307 study participants were included. Of them the magnitude of prolonged Prothrombin Time (PT) and Activated Partial Thromboplastin Time (APTT) were 68.08% and 63.51%, respectively. The presence of anaemia (AOR = 2.97, 95% CI: 1.26, 7.03), a lack of a vegetable feeding habit (AOR = 2.98, 95% CI: 1.42, 6.24), no history of blood transfusion (AOR = 3.72, 95% CI: 1.78, 7.78), and lack of physical exercise (AOR = 3.23, 95% CI: 1.60, 6.52) were significantly associated with prolonged PT. While the presence of anaemia (AOR = 3.02; 95% CI: 1.34, 6.76), lack of vegetable feeding habit (AOR = 2.64; 95% CI: 1.34, 5.20), no history of blood transfusion (AOR = 2.28; 95% CI: 1.09, 4.79), and a lack of physical exercise (AOR = 2.35; 95% CI: 1.16, 4.78) were significantly associated with abnormal APTT.

**Conclusion:**

Patients with liver disease had substantial coagulation problems. Being anemic, having a transfusion history, lack of physical activity, and lack of vegetables showed significant association with coagulopathy. Therefore, early detection and management of coagulation abnormalities in liver disease patients are critical.

## Background

Liver diseases are various disorders that can affect liver cells and impair normal liver function [1]. It is one of the world's most critical public health problems. The most common causes of the rising burden of liver disease are chronic viral hepatitis, mainly hepatitis B virus (HBV) and hepatitis C virus (HCV), alcoholic liver disease (ALD), and non-alcoholic fatty liver disease, caused by obesity, diabetes, autoimmunity, and hemochromatosis, damage from medication or chemicals are the other most common causes and types of liver disease [2, 3]. The liver has more than 5,000 separate bodily functions, including the synthesis of coagulation factor proteins to control bleeding within a damaged blood vessel and the production of blood coagulation inhibitors to prevent blood clots in normal circulation. Furthermore, the liver is involved in the reticuloendothelial system, which plays an important role in the clearance of active coagulation products. Liver disease affects both primary and secondary hemostasis by impairing the synthesis of all blood coagulation factors, activators, and inhibitors; which are essential to the blood coagulation pathway and fibrinolytic systems [4]. Consequently, patients with liver disease will suffer from the consequences of prolonged coagulation time, decreased clearance of activated factors, low platelet count, hyperfibrinolysis, and accelerated intravascular coagulation [5].

The PT and the APTT are both affected by cirrhosis because in liver disease there is reduced production of clotting factors and anticoagulants that are dependent on and independent of vitamin K [6]. The laboratory values of several coagulation tests are used to manage the bleeding problem in liver disease patients [7]. Up to 60% of chronic and active liver disease patients have prolonged PT, an independent predictor of poor survival in individuals with advanced cirrhosis [8]. In patients with cirrhosis, considerable amounts of fresh frozen plasma are necessary to enhance clotting factor levels [9]. Additionally, for patients with cirrhosis and abnormal coagulation screening tests, supplementation of vitamin K also corrects abnormal coagulation results [10].

Hemorrhagic complications due to liver disease patients are a major consequence and significant reason for intensive care unit admission, which varies from 15 to 61%, with 17–20% of cirrhotic patients experiencing a new commencement of substantial bleeding [11]. Around one‐third of patients with acute liver failure died from bleeding consequences [12]. Liver disease primarily causes upper gastrointestinal bleeding, portal hypertension caused by coagulation defects results in death in up to 2–6.7% of cases. Acute upper gastrointestinal bleeding is still a major cause of death in liver disease, accounting for up to 48% of cases [13]. Despite its consequences, there is a scarcity of information on the magnitude and associated factors of coagulation abnormalities among liver disease patients in the study area. Therefore, the current study aimed to assess the magnitude and associated factors of coagulation abnormalities among liver disease patients.

## Methods and materials

### Study design, area, and period

A hospital-based cross-sectional study was conducted from August to October 2022 at the University of Gondar Comprehensive Specialized Hospital (UoG-CSH). The hospital is located in Gondar town. Gondar is found at a distance of 727 km from Addis Ababa, the capital city of Ethiopia, in the northwest direction, and at a distance of 175 km from Bahir Dar, the capital city of the Amhara National Regional State. According to the 2015 report of the central statistical agency of Ethiopia, Gondar has a population of 323,900 [14]. The town has one public comprehensive specialized hospital, which is one of the oldest teaching hospitals in the country and provides health services for more than 7 million people in Gondar town and surrounding catchment areas [15].

### Operational definitions

#### Habit of drinking tea or coffee

Habitual tea/coffee drinkers are defined by tea/coffee consumption of 120 mL/day or more for at least 1 year [16].

#### Physical exercise

Participant who performs the daily active exercise for about 30 min a day [17, 18].

#### Habit of feeding vegetables

It is a consistent action to integrate a variety of vegetables into one's diet or meal plan [19].

#### Habit of feeding meat

It is the consumption of animal-derived proteins such as beef, poultry, pork, fish, or other meats as a primary source of nutrition in one's daily diet [20].

### Population, variables, and sampling techniques

Liver disease patients who were attending the UoG-CSH during the data collection period and who fulfilled the inclusion criteria were considered as a study population. Although the coagulation parameters (PT and APTT) were taken as dependent variables, the socio-demographic variables (age, gender, marital status, educational level, and residence), clinical variables (comorbidity, nutritional characteristics, blood transfusion history, smoking, habit of alcohol consumption, types of liver disease, and habit of physical exercise), and behavioural related variables (smoking, habit of alcohol consumption, and habit of physical exercise) were taken as independent variables. A total of 307 consecutively selected liver disease patients were included in this study. Study participants with a history of hereditary coagulation disease, critically ill patients, pregnant women, patients who took oral contraceptives, and patients who took drugs such as aspirin, heparin, and warfarin were excluded from the study.

### Data collection methods and data quality management

The socio-demographic, lifestyle, and nutritional data were collected using a pretested structured questionnaire through a face-to-face interview, and the clinical data were collected using a data extraction sheet from the patient’s medical charts. Coagulation tests were carried out by a *Genrui CA51* coagulation analyzer. To maintain the quality of the data, quality control testing was performed for each procedure. Furthermore, standard operating procedures were strictly followed. Training was given to all data collectors prior to the actual data collection. During the data collection period, there was close supervision by the investigators.

### Statistical analysis

Epidata version 3.1 software was used to enter, code, clean, and sort data. The data were then exported to STATA version 14.0 software for analysis. Frequencies, proportions, and summary statistics were used to summarize the data. Pearson rank chi-square assumption fulfillment was checked for categorical variables. Bivariable and multivariable logistic regression were used to determine factors associated with coagulation abnormalities. The Hosmer–Lemeshow goodness of fit test with a *p*-value greater than 0.5 was used to validate the model fitness assumption. Finally, the odds ratio with a 95% confidence interval was used to express the strength of the association. Variables with a *p*-value < 0.05 from the multivariable analysis were considered to have a significant association with the outcome.

## Results

### Socio-demographic and clinical characteristics of study participants

In this study, a total of 307 study participants were included. Of them, 220 (71.66%), 213 (69.38%), 181 (59.28%), and 143 (46.58%) were males, from rural residences, married, and unable to read and write, respectively. Besides, the mean age of the study participants was 38.38 ± 15.13 years, ranging from 6 to 82 years (Table 1).

**Table 1:** Socio demographic of Liver disease patients at UoG-CSH, Northwest Ethiopia, 2022. (*n* = 307)

Variable	Category	Frequency	Percent (%)
**Sex**	Male	220	71.66
	Female	87	28.34
**Age**	< 18 (children)	28	9.12
	18–45 (young adult)	188	61.24
	> 45 (old adult)	91	29.64
**Residence**	Rural	213	69.38
	Urban	94	30.62
**Educational level**	Unable to read and write	143	46.58
	Primary school	119	38.76
	Secondary school	26	8.47
	College &University	19	6.19
**Marital status**	Married	223	72.67
	Unmarried	84	27.36
**Occupation**	Farmer	177	57.65
	Housewife	33	10.75
	Merchant	23	7.49
	Government employee	21	6.84
	Private employee	19	6.19
	Other	34	11.70
**Religion**	Orthodox	284	92.51
	Muslim	17	5.54
	Protestant	6	1.95

From a total of study participants, 157 (51.14%), 37 (12.05%), 87 (28.34%), and 26 (8.47%) of study participants had chronic liver disease (CLD), acute liver disease, viral hepatitis, and ALD, respectively. Among viral hepatitis study participants, about 62/87 (71.26%) and 25/87 (28.74%) had HBV and HCV, respectively. On the other hand, about 86 (28.01%) of the study participants had taken medication rather than liver disease drugs. About 51 (16.61%), 39 (12.70%), and 33 (10.75%) were anemic, had a history of blood transfusions, and had heart disease, respectively (Table 2).

**Table 2:** Clinical characteristics of liver disease patients at UoG-CSH Northwest, Ethiopia, 2022 (*n* = 307)

Variable	Category	Frequency	Percent (%)
**Use of medication other than liver disease**	Yes	86	28.01
	No	221	71.99
**History of anemia**	Yes	51	16.61
	No	256	83.61
**History of tuberculosis**	Yes	12	3.90
	No	295	96.10
**Presence of DM**	Yes	13	4.20
	No	294	95.80
**Presence of cardiac disease**	Yes	31	10.10
	No	276	89.90
**History of blood transfusion**	Yes	39	12.70
	No	268	87.30
**History of surgery**	Yes	10	3.30
	No	297	96.70
**Presence of cancer**	Yes	8	2.60
	No	299	97.40
**Presence of hypertension**	Yes	21	6.80
	No	286	93.2
**Presence of HIV/AIDS**	Yes	6	2.0
	No	301	98.0
**Types of liver disease**	Acute liver disease	37	12.05
	CLD	157	51.14
	ALD	26	8.47
	Viral hepatitis	87	28.34
**Presence of viral hepatitis**	Yes	87	28.34
	No	220	71.66

Abbreviations: *ALD Alcoholic Liver Disease, CLD Chronic Liver Disease, HIV Human Immunodeficiency Virus, DM Diabetes Miletus*

### Nutritional and life style characteristics of study participants

About 269 (87.91%), 287 (93.49%), and 237 (77.1%) of the study participants had a habit of drinking tea/coffee, habit of feeding meat, and habit of vegetable, respectively. However, most of the study participants 262 (85.34%) had no habit of physical exercise, and about 11 (3.58%) had a smoking habit (Table 3).

**Table 3:** Lifestyle characteristics of liver disease patients at UoG-CSH Northwest, Ethiopia, 2022 (*n* = 307)

Variable	Category	Frequency	Percent (%)
**Tea or coffee drinking habit**	Yes	270	88.24
	No	37	11.76
**Meat feeding habit**	Yes	284	92.51
	No	23	7.49
**Vegetable feeding habit**	Yes	237	7.2
	No	70	22.8
**Alcohol drinking habit**	Yes	205	66.8
	No	102	33.2
**Cigarette smoking habit**	Yes	11	3.58
	No	296	96.42
**Physical exercise habit**	Yes	45	14.66
	No	262	85.34

### Magnitude of coagulation abnormalities

**Fig. 1:**
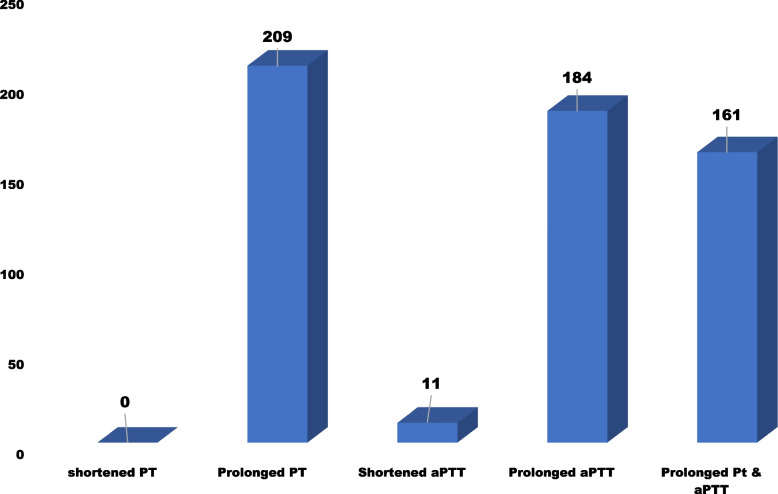
Magnitude of coagulation abnormalities among liver disease patients at the UoG-CSH, Northwest Ethiopia, 2022 (*n* = 307)

From the total study participants, 209 (68.08%, 95% CI: 62.8%, 73.3%) had a prolonged PT. On the other hand, 195 (63.51%; 95% CI: 58.1%, 68.9%) of the study participants had abnormal APTT. From the abnormal APTT, 184 (94.4%) patients had prolonged APTTs, and 11 (5.6%) had shorter APTTs. Furthermore, 161 (52.44%) study participants had both prolonged PT and abnormal APTT (Table 4, Fig. 1).

**Table 4:** Coagulation abnormalities tabulated with PT and APTT among liver diseased study participants at the UoG-CSH, Northwest Ethiopia, 2022 (*n* = 307)

Variable	Category	Prolonged PT		APTT	
		**Yes**	**No**	**Abnormal**	**Normal**
**Sex**	Male	146	74	138	82
	Female	63	24	57	30
**Age**	< 18 (children)	14	14	12	16
	18–45 (young adult)	131	57	120	68
	> 45 (old adult)	64	27	63	28
**Residence**	Urban	62	32	59	35
	Rural	147	66	136	77
**Occupation**	Farmer	127	50	115	62
	Housewife	23	10	22	11
	Merchant	11	12	17	6
	Government employee	13	8	10	11
	Private employee	14	5	12	7
	Other	21	13	19	15
**Educational level**	No education	101	44	93	50
	Primary school	76	42	76	43
	High school	20	6	16	10
	University & College	12	6	10	9
**Marital status**	Married	119	62	115	66
	Unmarried	90	36	80	46
**Use of medication other than liver disease**	Yes	58	28	57	29
	No	151	70	138	83
**History of anemia**	Yes	43	8	42	9
	No	166	90	153	102
**History of tuberculosis**	Yes	8	4	8	4
	No	201	94	187	108
**Presence of DM**	Yes	8	5	8	5
	No	201	94	187	107
**Presence of cardiac disease**	Yes	25	6	23	8
	No	184	92	172	104
**History of blood transfusion**	Yes	17	22	18	21
	No	192	76	177	91
**Have a history of surgery**	Yes	7	3	7	3
	No	202	95	188	109
**Presence of cancer**	Yes	6	2	4	4
	No	203	96	371	108
**Presence of hypertension**	Yes	15	6	12	9
	No	194	92	183	103
**Presence of HIV disease**	Yes	4	2	4	2
	No	205	96	202	110
**Consumption of tea or coffee**	Yes	183	87	171	99
	No	26	11	24	13
**Meat feeding habit**	Yes	192	92	179	105
	No	17	6	16	7
**Vegetable feeding habit**	Yes	151	86	140	97
	No	58	12	55	15
**Alcohol drinking habit**	Yes	139	66	126	79
	No	70	32	69	33
**Cigarette smoking habit**	Yes	7	4	6	5
	No	202	94	189	107
**Physical exercise habit**	Yes	22	23	22	23
	No	187	75	173	89
**Types of liver disease**	Acute liver disease	17	20	21	16
	CLD	108	49	92	65
	ALD	20	6	19	7
	Viral hepatitis	64	23	63	24
**Presence of virus hepatitis (HBV and HCV)**	Yes	53	34	50	37
	No	156	64	145	75

*Abbreviations: ALD Alcoholic Liver Disease, CLD Chronic Liver Disease, HIV Human Immunodeficiency Virus, DM Diabetes Miletus, Other includes student and no jobs*

### Factors associated with coagulation abnormalities

Bivariable and multivariable logistic regressions were performed to determine the association between PT abnormality and independent variables. Bivariable and multivariable logistic regressions were done for variables that fulfilled the chi-square assumption. The presence of anemia, physical exercise, a history of blood transfusion, the type of liver disease, the habit of feeding vegetables, and the frequency of feeding vegetables were associated with prolonged PT in bivariable analysis. Variables with a p-value of less than 0.25 in the bivariable analysis were selected for multivariable logistic regression. After multivariable logistic regression analysis, the history of anaemia (AOR = 2.97; 95% CI: 1.26, 7.03), the absence of a history of blood transfusion (AOR = 3.72; 95% CI: 1.78, 7.78), the absence of a vegetable feeding habit (AOR = 2.98; 95% CI: 1.42, 6.24), and the lack of physical exercise (AOR = 3.23; 95% CI: 1.60, 6.52) are significantly associated with prolonged PT (Table 5).

**Table 5:** Bivariable and multivariable logistic regression of PT among liver disease patients attending at the UoG-CSH, Northwest, Ethiopia, 2022 (*n* = 307)

Variables	Category	PT		Bivariable analysis		Multivariable
		**Prolonged PT (%)**	**Normal PT (%)**	**COR (95%CI)**	*P***-value**	**AOR (95%CI)**
Sex	Male	146 (66.36)	74 (33.64)	0.75 (0.43, 1.29)	.306	-
	Female	63 (72.41)	24 (27.59)	1		
Age	< 18 (children)	14(50.00)	14(50.00)	1		1
	18–45 (young adult)	131 (69.68)	57(30.32)	2.29(1.02, 5.13)	0.042	1.09(0.35, 3.38)
	> 45 (old adult)	64 (70.33)	27(29.67)	2.37(0.99, 5.63)	0.051	0.85(0.25, 2.84)
Residence	Rural	147(69.01)	66(30.99)	1.15(0.68, 1.92)	0.597	-
	Urban	62(65.96)	32(34.04)	1		
Occupation of study participants	Farmer	127(71.75)	50(28.25)	1.56(0.61, 3.99)	0.351	-
	Housewife	23(69.70)	10(30.30)	1.41(0.44, 4.47)	0.554	
	Merchant	11(47.83)	12(52.17)	0.56(0.16, 1.87)	0.351	
	Private employee	14(73.68)	5(26.32)	1.72(0.44, 6.63)	0.429	
	Other	21(61.76)	13(38.24)	0.99(0.32, 3.04)	0.992	
	Government employee	13(61.90)	8(38.10)	1		
Educational level	No education	101(69.23)	44(30.77)	1.03(0.37, 2.91)	0.943	-
	Primary school	76(64.71)	42(35.29)	0.84(0.29, 2.38)	0.752	
	High school	20(76.92)	6(23.08)	1.53(0.40, 5.81)	0.525	
	University & college	12(68.42)	6(31.58)	1		
Marital status	Unmarried	90(71.43)	36(28.57)	1.30(0.79, 2.13)	0.294	-
	Married	119(65.75)	62(34.25)	1		
Use of medication other than liver disease	Yes	58(67.44)	28(32.56)	0.96(0.56, 1.63)	0.881	-
	No	151(68.33)	70(31.67)	1		
History of anemia	Yes	43(84.31)	8(15.69)	2.91(1.31, 6.46)	0.009	2.97(1.26, 7.03)
	No	166(64.84)	90(35.16)	1		1
Presence of DM	Yes	9(69.23)	4(30.77)	1.05(0.31, 3.52)	0.927	-
	No	200(68.03)	94(31.97)	1		
Presence of cardiac disease	Yes	25(80.65)	6(19.35)	2.08(0.82, 5.25)	0.120	1.87(0.68, 5.15)
	No	184(66.67)	92(33.33)	1		1
History of blood transfusion	No	192(71.64)	76(28.36)	3.26(1.64, 6.49)	0.003	3.72(1.78, 7.78)
	Yes	17(43.59)	22(56.41)	1		1
Presence of hypertension	Yes	15(71.43)	6(28.57)	1.18(0.44, 3.15)	0.733	-
	No	194(67.83)	92(32.17)	1		
Tea or coffee drinking habit	No	26(70.27)	11(29.73)	0.97(0.46, 2.04)	0.955	-
	Yes	183(67.78)	87(32.22)	1		
Meat feeding habit	Yes	192(67.61)	92(32.39)	0.73(0.28, 1.93)	0.534	-
	No	17(73.91)	6(26.09)	1		
Vegetable feeding habit	No	58(82.86)	12(17.14)	2.75(1.40, 5.40)	0.003	2.98(1.42, 6.24)
	Yes	151(63.71)	86(36.29)	1		1
Alcohol drinking habit	Yes	139(67.80)	66(32.20)	0.96(0.57, 1.60)	0.884	-
	No	70(68.63)	32(31.37)	1		
Cigarette smoking habit	Yes	7(63.64)	4(36.36)	0.81(0.23, 2.84)	0.748	-
	No	202(68.24)	94(31.76)	1		
Physical exercise habit	No	187(71.37)	75(28.63)	2.60(1.37, 4.95)	0.003	3.23(1.60, 6.52)
	Yes	22(48.89)	23(51.11)	1		1
Types of liver disease	CLD	108(68.79)	49(31.21)	2.59(1.25, 5.37)	0.010	3.01(1.10, 8.21)
	ALD	20(76.92)	6(23.08)	3.92(1.28, 11.99)	0.017	4.97(1.27, 17.41)
	Viral hepatitis	64(73.56)	23(26.44)	3.27(1.46, 7.30)	0.004	3.56(1.19, 10.63)
	Acute liver disease	17(45.95)	20(54.05)	1		1
Presence of virus hepatitis (HBV and HCV)	Yes	53(60.92)	34(39.08)	0.63(0.38, 1.07)	0.092	0.74(0.42, 1.33)
	No	156(70.91)	64(29.09)	1		1

Abbreviation: * = *Significant Variable, ALD Alcoholic Liver Disease, APTT Activated Partial Thromboplastin Time, CLD Chronic Liver Disease, DMDiabetes millets, AOR,* *Adjusted odds ratio, CORCrude Odd Ratio, CI Confidence Interval, 1 reference group other includes student and have no jobs*

To determine the association between APTT abnormality and independent variables, both bi-variable and multi-variable binary logistic regression were done. The analysis includes independent variables that satisfy the chi square assumption. The presence of anemia, physical exercise, a history of blood transfusion, a type of liver disease, the feeding habits of vegetables, and age group were associated with APTT abnormalities in bivariable analysis. Variables with a *p*-value of less than 0.25 in the bivariable analysis were selected for multivariable logistic regression. After multivariable logistic regression analysis, young adult (18–45) (AOR = 5.22; 95%CI: 1.26, 21.63), old adult (> 45) (AOR = 5.49; 95%CI: 1.21, 24.82), presence of anemia (AOR = 3.02; 95% CI: 1.34, 6.76), lack of blood transfusion history (AOR = 2.28; 95%CI: 1.09, 4.79), lack of vegetable feeding habit (AOR = 2.64; 95%CI: 1.34, 5.20), and not doing physical exercise (AOR = 2.35; 95%CI: 1.16, 4.78) remained significantly associated with APTT abnormality ( \* MERGEFORMAT Table 6).

**Table 6:** Bivariable and multivariable logistic regression of abnormal APTT among liver disease patients attending at the UoG-CSH, Northwest, Ethiopia, 2022 (*n* = 307)

Variable	Category	APTT		Bivariable analysis		Multivariable analysis
		**Abnormal (%)**	**Normal (%)**	***P*-value**	**COR (95%CI)**	**AOR (95%CI)**
**Sex**	Male	138(62.73)	82(37.27)	0.567	0.85 (0.50, 1.44)	-
	Female	57(65.52)	30(34.48)		1	
**Age**	< 18 (children)	12(42.86)	16(57.14)		1	1
	18–45 (young adult)	120(63.83)	68(36.17)	0.037	2.35(1.05, 5.2)	5.22(1.26, 21.63)
	> 45 (old adult)	63(69.23)	28(30.77)	0.013	3 (1.25, 7.16)	5.49(1.21, 24.82)
**Residence**	Rural	136(63.85)	77(36.15)	0.856	1.03(0.62, 1.72)	-
	Urban	59(62.77)	35(37.23)		1	
**Occupation**	Farmer	115(64.97)	62(35.03)	0.125	2.04(0.82, 5.07)	1.84(0.66, 5.12)
	Housewife	22(66.67)	11(33.33)	0.168	2.20(0.71, 6.75)	1.59(0.46, 5.50)
	Merchant	17(73.91)	6(26.09)	0.078	3.11(0.87, 11.03)	3.61(0.94, 13.92)
	Private employee	12(63.16)	7(36.84)	0.326	1.88(0.53, 6.68)	1.63(0.41, 6.41)
	Other	19(55.88)	15(44.12)	0.551	1.39(0.46, 4.15)	3.21(0.74, 13.77)
	Government employee	10(47.62)	11(52.38)		1	1
**Educational level**	No education	93(65.03)	50(34.97)	0.295	1.67(0.63, 4.38)	-
	Primary school	76(63.87)	43(36.13)	0.351	1.59(0.59, 4.21)	
	High school	16(61.54)	10(38.46)	0.551	1.44(0.43, 4.77)	
	University& College	10(52.63)	9(47.37)		1	
**Marital status**	Unmarried	80(63.49)	46(36.51)	0.994	0.99(0.62, 1.60)	-
	Married	115(63.54)	66(36.46)		1	
**Use of medication other than liver disease**	Yes	57(66.28)	29(33.72)	0.531	1.18(0.70,1.99)	
	No	138(62.44)	83(37.56)		1	
**History of anemia**	Yes	42(82.35)	9(17.65)	0.003	3.14(1.46, 6.73)	3.02(1.34, 6.76)
	No	153(59.77)	102(40.23)		1	1
**Presence of DM**	Yes	8(61.54)	5(38.46)	0.880	0.91(0.29, 2.86)	-
	No	187(63.61)	107()36.39		1	
**Presence of heart disease**	Yes	23(74.19)	8(25.81)	0.197	1.73(0.75, 4.02)	1.48(0.61, 3.62)
	No	172(62.32)	104(37.68)		1	1
**History of blood transfusion**	No	177(66.04)	91(33.96)	0.018	2.26(1.15, 4.47)	2.28(1.09, 4.79)
	Yes	18(46.15)	21(53.85)		1	1
**Presence of hypertension**	Yes	12(57.14)	9(42.86)	0.531	0.75(0.30, 1.84)	-
	No	183(63.99)	103(36.01)		1	
**Tea or coffee drinking habit**	No	24(64.86)	13(35.14)	0.878	1.05(0.15, 2.17)	-
	Yes	171(63.33)	99(36.67)		1	
**Meat feeding habit**	Yes	179(63.03)	105(36.97)	0.532	0.74(0.29, 1.87)	-
	No	16(69.57)	7(30.43)		1	
**Vegetable feeding habit**	No	55(78.57)	15(21.43)	0.004	2.54(1.35, 4.75)	2.64(1.34, 5.20)
	Yes	140(59.07)	97(40.93)		1	1
**Alcohol drinking habit**	Yes	126(61.46)	79(38.54)	0.289	0.76(0.46, 1.25)	-
	No	69(67.65)	33(32.35)		1	
**Cigarette smoking habit**	Yes	6(54.55)	5(45.45)	0.531	0.67(0.20, 2.27)	-
	No	189(63.85)	107(36.15)		1	
**Physical exercise habit**	No	173(66.03)	89(33.97)	0.029	2.03(1.07, 3.84)	2.35(1.16, 4.78)
	Yes	22(48.89)	23(51.11)		1	1
**Type of liver disease**	CLD	92(58.60)	65(41.40)	0.838	1.07(0.52, 2.22)	0.53(0.18, 1.53)
	ALD	19(73.08)	7(26.92)	0.189	2.06(0.69, 6.11)	1.17(0.28, 4.81)
	Viral hepatitis	63(72.41)	24(27.59)	0.090	2(0.89, 4.46)	0.94(0.30, 2.95)
	Acute liver disease	21(56.76)	16(43.24)		1	1
**Presence of virus hepatitis (HBV and HCV)**	Yes	50(57.47)	37(42.53)	0.167	0.69(0.42, 1.16)	0.88(0.50, 1.55)
	No	145(65.91)	75(34.09)		1	1

Abbreviation: * = *Significant Variable, ALD Alcoholic Liver Disease, APTT Activated Partial Thromboplastin Time, CLD Chronic Liver Disease DM* *Diabetes millets, AOR* *Adjusted odds ratio, COR* *Crude Odd Ratio, CI*  *Confidence Interval, 1*  *the reference group, other* *Includes student and have no jobs*

## Discussion

Liver disease is a global public health problem that results in mortality and morbidity. It is one of the main causes of coagulopathy both in developed and developing countries [21]. Thus, the magnitude and associated factors of coagulation abnormalities in liver disease patients attending UoG-CSH were investigated in this study.

The findings of this study showed that the overall prevalence of prolonged PT and abnormal APTT test results was 68.08% (95% CI: 62.8%, 73.3%) and 63.51% (95% CI: 58.1%, 68.9%), respectively. This value represents a high public health problem for patients with liver disease. The magnitude of abnormal PT and APTT in this study is significant. This high result is due to liver disease, which reduces the production of clotting factors, particularly vitamin K-dependent factors. Hence, patients with liver disease experience a range of hemostatic problems, including reduced production of clotting factors and coagulation inhibitor proteins [5, 6]. In contrast, the shorter APTT finding might be associated with inflammation, which may initiate clotting and decrease the activity of natural anticoagulant mechanisms. Additionally, inflammatory cytokines are also the major mediators involved in coagulation activation [22]. The prolonged PT in this study was consistent with a study reported by Bohania N et al. 68.33% [23], *and Yatish PA *et al*. 63%* [24]. In contrast, the finding of this study was lower than a study by *Chetali Rupela *et al*.* 86.6% [25], *Parashat Patel *et al*.* 85% [26], and *Siddiqui SA *et al*.* 88% [27]. The differences could be due to the variety of the study population or the way the tests were done. However, it is higher than that of *Garg RP *et al. 4.90% [28], *Shobhaha P *et al*.* 42.22% [29], *Bhatia G *et al*.* 62% [30]. The possible reason for the high coagulation abnormality in this study may be associated with the presence of other comorbidities in this study; mainly, 16.61% were anaemic and 10.75% had cardiac disease; additionally, 86 (28.01%) patients had treatment other than liver disease drugs.

The magnitude of abnormal APTT in this study was 63.51% (95% CI: 58.1%, 68.9%); it was in line with a study conducted by Bohania et al., which found a 61.67% prevalence of abnormal APTT [23]. In contrast, the magnitude of the abnormal APTT was lower than that of Chetali R. et al. (82.2%) [25], *Siddiqui SA *et al*. (*71%) [27]. The differences could be in the study population or the way the tests were done, but this study finding was higher than a study reported by *Shobhaha. P *et al*. (*26.66%) [29], *Bhatia G *et al*.* (39.3%) [30], *Yatish PA *et al*. (*56%) [24], *Parashat P. *et al.,* (*52%) [26]. The possible reason may be associated with the presence of other comorbidities; mainly, 51 (16.61%) patients were anaemic and 33 (10.75%) had heart disease; additionally, 86 (28.01%) patients had treatment other than liver disease drugs. Additionally, possible reasons for the difference might be associated with differences in the study population, geographical variability, or the way the test was done.

Anemia was statistically associated with prolonged PT and abnormal APTT. In the current study, study participants has history of anaemia were 2.97 times (95% CI: 1.26, 7.03) more likely to have prolonged PT and 3.02 times (95% CI: 1.34, 6.76) more likely to have APTT abnormalities when compared with those without anemia. This is due to an anaemic patients' delayed response in the initiation of the coagulation cascade [31].

Types of liver disease were statistically associated with prolonged PT. Study participants with CLD had a 3.01-fold (95% CI: 1.10, 8.21) higher likelihood of being associated with prolonged PT; study participants with ALD had a 4.97-fold (95% CI: 1.27, 17.41) higher likelihood of being associated with prolonged PT; and study participants with viral hepatitis had a 3.56-fold (95% CI: 1.19, 10.63) higher likelihood of being associated with prolonged PT when compared to acute liver disease. Scientific explanations suggest that the liver plays a central role in the clotting process and is invariably associated with coagulation disorders due to decreased synthesis of clotting and inhibitory factors. Additionally, liver disease causes decreased synthesis of clotting factors, mainly vitamin K-dependent factors [6].

In this study, the young adult age (18–45) class has a 5.22-fold (95% CI: 1.26, 21.63) higher likelihood of being associated with abnormal APTT, whereas study participants in the old adult (> 45) age class have a 5.49-fold (95% CI: 1.21, 24.82) higher likelihood of being associated with abnormal APTT when compared with children (< 18). According to research, as one gets older, the liver's proliferative and metabolic functions may decline [32]. Since the regenerative capacity of the liver correlates with liver function [33].

Physical exercise was statistically associated with coagulation abnormalities. Study participants who did not do physical exercise had 3.23 times (95% CI: 1.60, 6.52) more likely associations with prolonged PT and 2.35 times (95% CI: 1.16, 4.78) more likely associations with APTT abnormalities when compared with those who did. This is due to the fact that physical exercise enhances the activation of both the coagulation and fibrinolytic cascades and increases the activity of several components of the coagulation cascades [34]. Additionally, it is suggested that short-term exercise activates blood coagulation, enhances blood fibrinolysis, and maintains the delicate balance between clot formation and clot dissolution [35].

The history of blood transfusion has been statistically associated with coagulation abnormalities. A study participant without a history of blood transfusion has a 3.72-fold (95% CI: 1.78, 7.78) more likely association with prolonged PT and a 2.28-fold (95% CI: 1.09, 4.79) more likely association with an APTT abnormality when compared with a participant with a history of blood transfusion. A scientific explanation suggests blood transfusions reintroduce blood clotting elements into the patient's blood. Additionally, whole blood contains approximately 150 mL of plasma, which provides the patient with non-labile clotting factors [36].

Vegetable feeding habits were statistically associated with coagulation abnormalities. Study participants with a lack of vegetable feeding habits had 2.98 times (95% CI: 1.42, 6.24) more likely associated with prolonged PT and 2.64 times (95% CI: 1.34, 5.20) more likely associated with APTT abnormality when compared with study participants who have a vegetable feeding habit. Scientific suggestions are that vitamin K and dependent coagulation factors such as FII, FVII, FIX, and FX are essential for regulating blood coagulation and comprise the coagulation factors. Vitamin K is found in the diet in two bioactive forms: phylloquinone (vitamin K1) and menaquinones (vitamin K2), both of which are abundant in leafy green vegetables [37, 38].

### Strength and limitation of the study

This study has its strengths and limitations. The strength of this study is that it is the first study on the determination of the magnitude and associated factors of coagulation abnormalities in liver disease patients in Ethiopia. However, the limitation of this study was that we could not perform parasitic infection screening, which may interfere with the finding of the study.

### Conclusion on recommendation

Coagulation abnormalities in liver disease were identified as a major public health issue. About half of liver disease patients had coagulation abnormalities (prolonged PT and abnormal APTT). Prolonged PT was associated with the presence of anemia, an absence of transfusion history, a habit of physical exercise, types of liver disease, and vegetable feeding habits. Abnormal APTT was associated with the presence of anemia, the absence of transfusion history, a habit of physical exercise, increased age, and vegetable feeding habits. Based on the high prevalence of coagulation abnormalities in patients with liver disease, it is recommended that healthcare providers should regularly monitor and assess coagulation function in these patients. This can help identify any potential bleeding and allow for timely intervention to prevent complications.

## Data Availability

All the data supporting these findings are contained within the manuscript.

## Abbreviations

ALD: Alcoholic Liver Disease
APTT: Activated Partial Thromboplastin Time
CLD: Chronic Liver Disease
HBV: Hepatitis B virus
HCV: Hepatitis C virus
PT: Prothrombin Time
UoG-CSH: University of Gondar Comprehensive Specialized Hospital
